# Using Coordinated Visual and Verbal Cues in Complex Multimedia Materials to Improve Tactical Learning in Soccer

**DOI:** 10.3390/ijerph19063365

**Published:** 2022-03-12

**Authors:** Nourhen Mezghanni, Ghazi Rekik, Zachary J. Crowley-McHattan, Yosra Belkhir, Rayda Ben Ayed, Atyh Hadadi, Turki Mohsen Alzahrani, Cheng-Deng Kuo, Yung-Sheng Chen

**Affiliations:** 1 Department of Sport Sciences, College of Education, Taif University, Taif 21944, Saudi Arabia; nsmezghanni@tu.edu.sa (N.M.); atyhhadadi@gmail.com (A.H.); tm.alzahrani@tu.edu.sa (T.M.A.); 2 Tanyu Research Laboratory, Taipei 112, Taiwan; cdkuo23@gmail.com; 3 Research Laboratory—Education, Motricity, Sport and Health (LR19JS01), High Institute of Sport and Physical Education, Sfax University, Sfax 3000, Tunisia; belkhir.ysr@gmail.com; 4 Discipline of Sport and Exercise Science, Faculty of Health, Southern Cross University, Lismore 2480, Australia; zac.crowley@scu.edu.au; 5 Department of Physical Education, Al-Udhailiyah Primary School for Girls, Kuwait City 085700, Kuwait; 6 Laboratory of Molecular and Cellular Screening Processes, Center of Biotechnology of Sfax, Sfax University, Sfax 3000, Tunisia; raydabenayed@yahoo.fr; 7 Department of Medicine, Taian Hospital, Taipei 104, Taiwan; 8 Department of Medical Research, Taipei Veterans General Hospital, Taipei 112, Taiwan; 9 Department of Exercise and Health Sciences, University of Taipei, Taipei 111, Taiwan; 10 Exercise and Health Promotion Association, New Taipei City 241, Taiwan

**Keywords:** diagram, arrows, narration, game actions, visual cue, verbal cue, tactical learning, soccer

## Abstract

This study aimed to explore whether the use of coordinated visual and verbal cues in narrated diagrams would support novices in learning soccer scenes. Eighty female university students (M_age_ = 20 years, SD = 1.2) in physical education (PE) were randomly exposed to four multimedia material versions: (a) simple without cues, (b) simple with cues, (c) complex without cues, and (d) complex with cues. In the non-cued versions, students learned the evolution of soccer scenes via arrow-based diagrams accompanied by oral explanations. In the cued versions, game actions in narrations were spoken with a louder accent (verbal cueing), while at the same time, the corresponding arrows turned red in diagrams (visual cueing). After studying one of the four versions, participants were asked to complete immediate and delayed recall–reconstruction tests, and to indicate their attitudes towards instructional materials. The results demonstrate the instructional benefits of using coordinated dual-modality cues in the complex multimedia material, in terms of immediate (*p* = 0.03, *d* = 0.53) and delayed (*p* = 0.02, *d* = 0.85) recall performances. The findings encourage soccer teachers to verbalize game actions with a louder accent, while simultaneously coloring the corresponding arrows in the diagram when explaining complex game situations for novices.

## 1. Introduction

In team sports such as soccer, tactical learning refers to the ability of an individual student or player to effectively memorize/understand the strategies and tactics depicted in game situations [1]. To explain offensive/defensive playing systems, most soccer instructors rely heavily on diagrams as suitable didactical tools [1]. With these instructional materials, soccer teachers/coaches communicate the essential components of the game situations through two mediums. The first medium consists of the provision of cross symbols to depict visuo-spatial information (i.e., the players and the ball). The second compelling medium consists of the provision of arrows to depict temporal information (i.e., game actions). As a class of basic diagrammatic elements [2], arrows are considered to be a crucial component of soccer diagrams for conveying the essential game actions occurring on the pitch (e.g., passing, dribbling, shooting, etc.). On the other hand, when explaining soccer playing systems, the use of diagrams is usually accompanied by a coherent/structured oral speech (i.e., narration) that include some words related to motor behaviors (e.g., player ➆ *moves* inside the penalty area, player ➈ *dribbles* or *shoots*) [3]. These specific, spoken words can be used to support the learning process due to an automatic activation of the mirror-neuron system [4]. 

In accordance with the cognitive theory of multimedia learning (CTML) [5,6,7], a narrated diagram is processed by two separate cognitive subsystems located in working memory (WM): a verbal system for auditory/verbal information, and an imagery system for visual/pictorial information. In this framework, Mayer [6] suggested that a dual-mode encoding of information is more efficient for learning than a single-mode encoding, because it enlarges the effective size of WM capacity. This multimedia learning effect has been reported in several studies showing that cognitive load and learning were enhanced when the diagram is accompanied by an oral explanation [8,9,10]. For example, using the dual-task measurement of cognitive load in different multimedia learning environments [11], the results of two previous studies conducted by Brünken and his colleagues [12,13] show that students working with the diagrams acquired less knowledge than students working with the narrated diagrams. However, learning from such multimedia material (i.e., narrated diagrams) could be a challenging task, especially for novice learners, due to the limited capacity of both cognitive subsystems of WM to simultaneously process information through two sensory modes [7,14]. Indeed, following the CTML, to achieve meaningful multimedia learning, learners should successfully complete three cognitive processes: (i) the selection of visual information to be processed by the imagery system and the selection of auditory information to be processed by the verbal system; (ii) the organization of the selected auditory information into a verbal mental representation, as well as the organization of the selected visual information into a visual mental representation; (iii) the integration of verbal and visual mental representations with each other, and with relevant knowledge stored in long-term memory.

To support the cognitive processing of narrated diagrams, several studies have suggested an advantage for “*the cueing technique*” in learning achievement and some subjective experiences (see [15,16,17]). A group of studies have shown that the incorporation of visual cues in diagrams (e.g., via coloring or flashing) enhances learning performance and motivation [3,18,19]. Another group of studies have shown that using verbal cues in narrations (e.g., by varying the volume or intonation of the speaker’s voice) can improve learning outcomes [20,21]. We might infer from these studies that providing single-modality cues (i.e., either visual or verbal cues) can guide learners’ attention to effectively select some crucial information elements from diagrams or narrations, thereby improving the multimedia learning process [3,19,20]. More recently, Xie et al. [22] found that using coordinated visual and verbal cues in a narrated diagram resulted in optimal learning outcomes (compared with visual cues, verbal cues, or no cues). The authors suggested that providing visual and verbal cues in a coordinated manner is the most valuable technique to guide learners’ attention, thereby promoting the cognitive process of integration of both visual and auditory information in WM.

While many studies have investigated cueing effects in learning from narrated diagrams, surprisingly little is known how such instructional design techniques could affect tactical learning from these multimedia materials. To the best of our knowledge, there is only one scientific work that has been interested in this topic [3], which indicates that adding visual cues in narrated soccer diagrams was effective for improving motivation and tactical learning among male novice players. In the current study, we try to determine how the use of coordinated visual and verbal cues in narrated soccer diagrams could affect tactical learning and attitudes in female novice learners. No previous research has investigated this issue, which is a significant contribution of the current study. It was hypothesized that using coordinated dual-modality cues in multimedia materials (i.e., simple and/or complex) would elicit more positive attitudes to learn the scenes of play (expressed in terms of attention, motivation, enjoyment, interestingness, enjoyment, and self-efficacy) among students. It was also hypothesized that using coordinated dual-modality cues in multimedia materials would further support the short- and long-term memories of students, thereby enhancing their immediate and delayed recall performances when reproducing the evolution of the playing systems (through paper and pencil tests).

## 2. Materials and Methods

### 2.1. Trial Design

The experiment used a 2 × 2 factors between-subjects design, with a condition (with cues vs. no cues) and a type of material (simple vs. complex). Participants were randomly assigned to one of the four resulting experimental conditions, with 20 participants in each condition. The sample size was based on a priori power analysis using G*Power software (Version 3.1, Düsseldorf, Germany) [23], with an alpha level of 0.05, the power of 0.80, and the effect size of 0.46 (derived from a previous study [24]). Dependent variables included immediate and delayed recall performances, as well as attitudes towards instructional materials.

### 2.2. Participants

Participants were a total of 80 female PE students recruited from the Taif University (Taif, Saudi Arabia). The mean age was 20 years (standard deviation = 1.2 years). The convenience sample was used in this study. The inclusion criteria included (i) female students enrolled at the Taif University, (ii) aged between 20 and 22 years, and (iii) no experience in playing soccer or any other team ball sports in semi-professional and/or professional clubs (to avoid the potential effects of transfer across sports [25]). The exclusion criteria included (i) visual and auditory impairment, and/or (ii) any cognitive disabilities. All participants gave their consent to participate in this study by signing a consent form. Approval for this project was guaranteed by the Taif University (project number: TURSP-2020/184). 

### 2.3. Apparatus and Materials

#### 2.3.1. Computerized Materials

The apparatus consisted of a computer (HP Elite Dragonfly Max Laptop, Beijing, China) placed at a distance of 30 cm from the participant (with a viewing angle of approximately 45°), and headphones (Sony MDR-ZX110, Tokyo, Japan).

For all experimental conditions, the participants interacted with the computer-based learning environment, which comprised the following phases: (a) a questionnaire requesting students to report their age and proficiency in team sports; (b) an instructional session that provided an explanation of the meaning of arrow symbols used in soccer diagrams; and (c) a study phase. The study phase consisted of a projection (on a PowerPoint page) of one of the four multimedia material versions, and the participant was asked to memorize, as precisely as possible, the evolution of the scene of play.

The two diagrams and the two narrations (depending on the amount of information to be processed) were developed by a qualified soccer coach with over 16 years’ experience. The simple diagram and/or narration represented a soccer scene that included four players (#13-center midfield, #10-attacking midfield, #7-right winger, and #11-left winger) who carried out a coherent tactical combination which was composed of 8 game actions. The complex diagram and/or narration represented a soccer scene that included eight players (#8-defensive midfield, #2-right wing-back, #3-left wing-back, #13-center midfield, #10-attacking midfield, #7-right winger, #11-left winger, and #9-center forward) who carried out a coherent tactical combination which was composed of 14 game actions. Note that the narration was presented by a female voice in Arabic.

**Figure 1:**
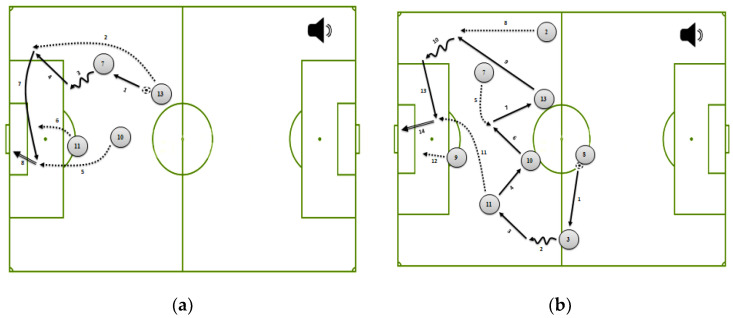
Simple (**a**) and complex (**b**) multimedia materials without cues.

Afterwards, two versions of the simple and/or complex multimedia material (i.e., combination of diagram and narration) were developed via PowerPoint software. The first version showed the evolution of the game situations without cues (Figure 1). In other words, students learned the evolution of soccer scenes via arrow-based diagrams accompanied by oral explanations.

**Figure 2:**
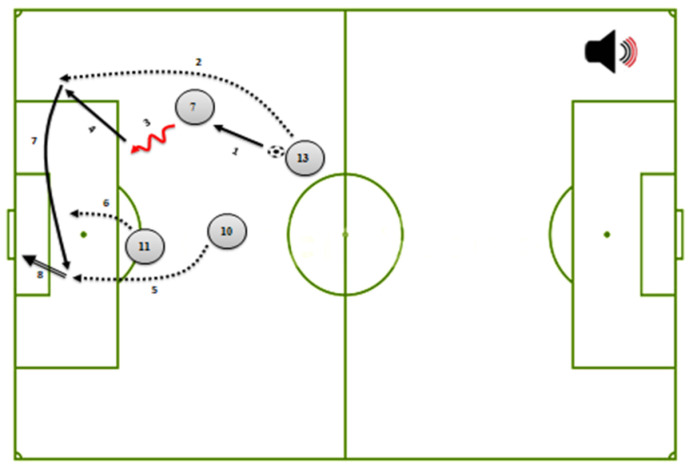
Simple multimedia material by coordinated dual (visual and verbal) cueing of the third game action.

The second version described the evolution of soccer scenes with a combination of visual and verbal cues. For verbal cues, the amplitude of keywords describing the game actions in the narration was increased by 20 dB (via Adobe Audition CS5 software), while keeping the rest of the narrated words at conventional levels. For visual cues, arrows representing the game actions on the screen were colored in red when corresponding keywords were mentioned in the narration, and then turned black. To create the coordinated dual cue conditions, both types of cues were added, with verbal cues and the corresponding visual cues appearing simultaneously. In other words, when the narrator verbalizes a game action with a louder accent, the corresponding arrow synchronously turned red in the diagram. A schematic representation for the simple multimedia material with coordinated dual cueing of the third game action is shown in Figure 2. Table 1 presents the keywords and their corresponding arrow symbols that are typically used to convey game actions in soccer [1]. Note that all computer-based versions was system-paced rather than self-paced to avoid the potential benefits of interactivity [26,27].

#### 2.3.2. Paper and Pencil Materials

**Figure 3:**
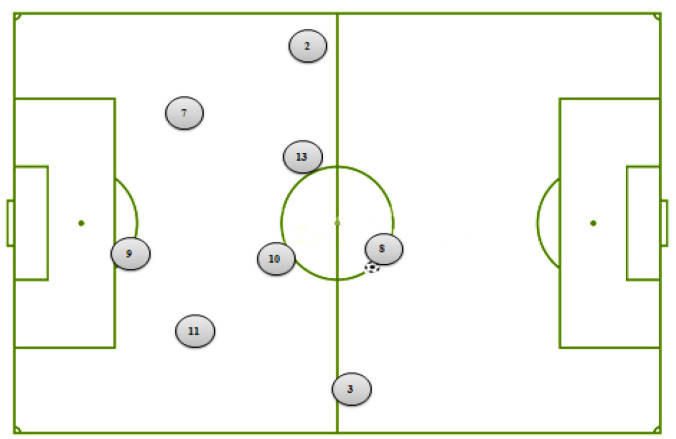
Recall–reconstruction test (immediate or delayed) used for the complex multimedia material.

Three paper and pencil materials were used to measure dependent variables: (1) An attitudinal questionnaire including five items (attention, motivation, enjoyment, interestingness, enjoyment, and self-efficacy) [28]. Responses to these questions ranged from 1 (*not at all*) to 9 (*extremely*). (2) The immediate recall–reconstruction test; and (3) the delayed recall–reconstruction test. The two recall–reconstruction tests were based on the reconstruction paradigm for the experimental analysis of the perceptual factors in cognitive processing [29]. During these two tests, the participants had to reproduce the evolution of the playing system as accurately as possible on a paper sheet that included a schematic of the soccer field and the players’ positions (Figure 3). More specifically, participants were instructed to use numbered arrows as well as the corresponding keywords to indicate all game actions. The participants were given one point for each correct response and no points for each incorrect response. Scores for simple material ranged from 0–8 points, while scores for complex material ranged from 0–14 points.

Two other interfering paper and pencil materials were used (but not to measure dependent variables) during the experimental procedure before the administration of the delayed recall–reconstruction test: (1) the card rotations test [30], and (2) the paper folding test [30]. These were true–false tests which each included two parts of 10 questions, in which the students were asked to see similarities and differences between the shapes (in the card rotations test), or to imagine the folding and unfolding of pieces of paper (in the paper folding test).

### 2.4. Procedure

The experiment was run per group of five students under laboratory conditions. In a random order, each student was tested individually with the experimenter observing a session that lasted approximately 30 min. The student was provided with a laptop, headphones, and an envelope containing the required paper and pencil tests. First, the experimenter described the study objectives, and the participant read and signed an informed consent form for participation. Next, the experimenter instructed the student to interact with the computer-based learning environment comprising the three phases mentioned above. Note that the learning material was shown twice during the test phase (following previous sporting studies [31,32]), and the order of exposure to the computer-based versions was counterbalanced among participants to control for ordered effects across conditions. Once the study phase was over, the student was given 2 min to complete the immediate recall–reconstruction test. Afterwards, there was about a 20 min break, during which the participant was instructed to perform three interfering tasks: 7 min to complete the card rotation test [30], 7 min to perform the paper folding test [30], and 5 min to count backwards by 3 from 999 [33]. These tasks were used to reduce the possibility that recall accuracy could occur as a result of short-term memory. Immediately after performing the interfering tasks, the student was given 2 min to complete the delayed recall–reconstruction test by reconstructing the same soccer scene that had previously been presented in the study phase. Finally, the student was given 1 min to respond to the 5-item attitudinal questionnaire. During each test, the time was controlled by the researcher using a handheld stopwatch. 

### 2.5. Statistical Analyses

The normality of the distribution was verified by the Shapiro–Wilk test. A two-way analysis of variance (ANOVA) was conducted to explore between-conditions differences. Mean and SD (standard deviation) values were determined for each dependent variable. An alpha level of 0.05 was used in reporting all statistical tests. The qualitative magnitudes were reported as partial eta squared ($n_{p}^{2}$) and Cohen’s d (*d*) for post hoc comparisons.

## 3. Results

### 3.1. Recall Performances

Means and (standard deviations) of immediate and delayed recall performances as a function of all experimental conditions are presented in Table 2.

Regarding the immediate recall performances, ANOVA showed that the main effect of condition (*F* (1.19) = 3.15, *p* = 0.09, $n_{p}^{2}$ = 0.14), and type of material (*F* (1.19) = 2.63, *p* = 0.12, $n_{p}^{2}$ = 0.12) was non-significant. The interaction between these two factors was also statistically non-significant (*F* (1.19) = 2.05, *p* = 0.16, $n_{p}^{2}$ = 0.10). A follow-up analysis revealed that participants in both conditions performed similarly (*p* > 0.05, *d* = 0.27) when the multimedia material was simple. However, participants in the with-cues condition performed significantly better than those in the no-cues condition (*p* = 0.03, *d* = 0.53) when the multimedia material was complex.

Concerning the delayed recall performances, ANOVA showed a significant main effect of condition, (*F* (1.19) = 12.96, *p* = 0.001, $n_{p}^{2}$ = 0.40). However, analysis showed that the main effect of type of material (*F* (1.19) = 1.71, *p* = 0.20, $n_{p}^{2}$ = 0.08), and condition × type of material interaction (*F* (1.19) = 1.65, *p* = 0.21, $n_{p}^{2}$ = 0.08) was non-significant. A follow-up analysis revealed that participants in both conditions performed similarly (*p* > 0.05, *d* = 0.51) when the multimedia material was simple. However, participants in the with-cues condition performed significantly better than those in the no-cues condition (*p* = 0.02, *d* = 0.85) when the multimedia material was complex.

### 3.2. Attitudes

The reliability and validity of the attitudinal questionnaire designed for this study were verified (Table 3). ANOVA on overall attitudes showed a non-significant main effect of condition (*F* (1.19) = 0.30, *p* = 0.59, $n_{p}^{2}$ = 0.01), and a non-significant main effect of type of material (*F* (1.19) = 0.17, *p* = 0.68, $n_{p}^{2}$ = 0.01). There was also a non-significant main effect of interaction between these two factors (*F* (1.19) = 2.11, *p* = 0.16, $n_{p}^{2}$ = 0.10). In each of the individual subscales, analysis indicated that learners in both conditions reported the same level of attention, motivation, self-efficacy, interestingness, and enjoyment (*p* > 0.05), whatever the type of the multimedia materials. Means and standard deviations of subjective-rating measurements as a function of all experimental conditions are given in Table 4.

## 4. Discussion

The current study was conducted to explore the potential instructional effectiveness of the dual-cueing technique in learning soccer scenes from multimedia materials (simple and complex). It was hypothesized that using coordinated visual and verbal cues in narrated diagrams (to highlight game actions) would improve learner’s attitudes and recall performances. This prediction was partially supported. 

On the one hand, considering the results of the attitudinal questionnaire, it was shown that participants reported similar reactions towards the different versions of multimedia equipment (i.e., either with or without cues), which could contradict the findings of some previous studies showing the affective benefits (e.g., in term of motivation) of adding visual cues when learning from narrated diagrams [3,18]. Clearly, it can be determined (in our study) that all students appreciated the instructional materials which were used, either with or without highlighting game actions through a combination of visual and verbal cues. In fact, the results indicate that the use/non-use of cues in the simple and/or complex multimedia material assisted students in developing their self-efficacy and holding their attention, which encouraged them to be more engaged/motivated in the learning process. According to Xie et al. [22], when explaining important knowledge to students, teachers tend to highlight the essential visual elements on display, while temporarily changing his/her voice properties to stress the corresponding content that is being described. Therefore, a plausible explanation for our non-significant results between conditions is that the study sample was familiarized (during regular lecture classes) with the dual-cueing technique when learning about other theoretical topics (e.g., anatomy or physiology) from multimedia materials.

On the other hand, considering the results of the two recall–reconstruction tests, it was found that the effectiveness of the dual-cueing technique depends heavily on the type of multimedia material. In the case of a simple material, highlighting game actions through coordinated visual and verbal cues was unnecessary and does not provide any obvious added value for tactical learning: participants achieved the same level of immediate and delayed recall performances. However, in the case of a complex material, highlighting game actions through coordinated visual and verbal cues was effective for improving tactical learning: participants achieved a higher level of immediate and delayed recall performances. First, these results could fit with some studies carried out in either team sports or in other instructional domains, indicating that instructional design techniques (e.g., employing self-control, decreasing the presentation speed) are only effective with the increase in the complexity of instructional materials [34,35,36,37,38]. For example, Rekik et al. [1] showed that a sequential presentation of information (either with or without tracing) improves tactical learning solely when processing complex soccer diagrams. Second, the obtained results remain closely related to the limited capacity assumption of CTML [5,7], with the proviso that a cueing technique may represent significant instructional benefits only during complex multimedia learning situations. In fact, simple multimedia materials lead to easier learning because learners have to consume fewer WM resources to deal with a small amount of visual and verbal information. Thus, learners may effectively succeed in the three cognitive processes (i.e., selection, organization, and integration) required for learning soccer scenes from narrated diagrams without cues. Contrariwise, dealing with complex multimedia materials could be a very challenging task for novice learners due to their limited WM capacity. Challenges arise from the large amount of visual and auditory information to be simultaneously selected and processed by the two separate cognitive subsystems (verbal and imagery systems) located in WM (i.e., disruption of the selection cognitive process). As a result, learners will easily fail to build structural relations among selected auditory information (to construct a verbal mental representation) and selected visual information (to construct a pictorial mental representation) (i.e., disruption of the organization cognitive process). Consequently, learners will not succeed in building connections between verbal and visual mental representations, and between information in WM and associated knowledge stored in long-term memory (i.e., disruption of the integration cognitive process). Under such circumstances, providing coordinated visual and verbal cues may reduce these demanding cognitive processes through three potential advantages: (a) the attention-guidance advantage, (b) the spatial-matched processing advantage, and (c) the temporal-synchronized processing advantage [22]. The first potential advantage helps learners in selecting (through visual cues) relevant visual information elements from the screen (arrows representing game actions, in our case) to be processed by the imagery system in WM, and in selecting (through verbal cues) crucial auditory information elements from the narration (keywords representing game actions, in our case) to be processed by the verbal system in WM. The two other potential advantages (b and c) are likely to be efficacious to promote the cognitive process of integrating corresponding visual and verbal information elements in WM as required in the CTML [22,39]. Indeed, while the spatial-matched processing advantage makes learners able to process the visual information elements that are spatially matched with the auditory information elements, the temporal-synchronized processing advantage helps learners in processing (synchronously) both the visual elements and the relevant spoken words. These ideas were supported by previous scientific works focused on cross-modal integration, showing that audio–visual integration is related to temporal synchronization [40], and semantic congruency/matching [41].

The present experiment offers insight into the instructional effectiveness of using coordinated dual-modality cues in soccer multimedia materials. However, some limitations should be mentioned. Firstly, findings remain valid only for learners with limited prior knowledge. It is unclear whether the current results would be replicated with more knowledgeable learners. Indeed, in accordance with the expertise reversal effect [42], instructional design techniques that are effective for novice learners can be the reverse for more experienced learners and become ineffective. In this vein, it has been established that learners’ prior knowledge has a significant moderating effect on the effectiveness of the cueing technique in multimedia learning [3,14,43]. Therefore, further studies are needed to test whether the effects of using coordinated visual and verbal cues in narrated soccer diagrams would also be affected by such kinds of individual differences. Secondly, the generalization of our results’ interpretation is limited by the number and gender of participants. Additional research should perhaps explore the pattern of our results with a larger sample size while taking into consideration the effect of students’ gender. Thirdly, this study suffers from low ecological validity as learning performances were obtained under laboratory conditions (through paper and pencil tests). It would be worthwhile in future studies to collect learning indicators (motor behaviors) in more realistic circumstances (e.g., via a game performance task carried out on the pitch). Lastly, it may be an interesting path for future research to use objective measures in order to explore how a coordinated cueing of visual and auditory information may affect brain and cognitive mechanisms. 

## 5. Conclusions

The current study demonstrated important practical implications for PE teachers using multimedia materials to communicate/explain tactical combinations of play. More specifically, the findings encourage soccer teachers to verbalize game actions with a louder accent while simultaneously coloring the corresponding arrows in the diagram when explaining complex playing systems for novices.

## Figures and Tables

**Table 1:** Keywords and their corresponding arrow symbols used to depict game actions in soccer.

Keywords	Arrow Symbols
Shot	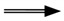
Dribble	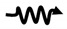
Pass	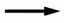
Move	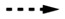

**Table 2:** Means and (standard deviations) of immediate and delayed recall performances as a function of all experimental conditions.

Measurement Items	No Cues		with Cues	
	Simple	Complex	Simple	Complex
Immediate recall performances	2.65 (1.81)	3.3 (2.79)	3.25 (2.55)	5 (3.61) *
Delayed recall performances	2.35 (1.73)	2.6 (1.85)	3.45 (2.54)	4.95 (3.43) *

* Significant difference compared to no-cues condition (*p* < 0.05).

**Table 3:** Reliability and validity of the 5-item attitudinal questionnaire.

Measurement Items	Component (Factor Loading) Attitudes	Cronbach’s Alpha Coefficient (α)
Enjoyment	0.906	0.745
Attention	0.497	
Motivation	0.867	
Interestingness	0.690	
Self-efficacy	0.518	
Eigen value	2.562	
Variance%	51.230	
Cumulative%	51.230	

Kaiser–Mayer–Olkin = 0.681; Bartlett’s test result χ^2^ = 136,732; (df = 10, *p* = 0.000).

**Table 4:** Means and (standard deviations) of subjective-rating measurements as a function of all experimental conditions.

Measurement Items	No Cues		with Cues	
	Simple	Complex	Simple	Complex
Overall attitudes	6.97 (1.25)	6.4 (1.62)	6.71 (1.3)	7 (1.45)
Enjoyment	7.05 (1.85)	6.1 (2.31)	7.3 (1.59)	7.5 (2.12)
Attention	6.95 (1.64)	7.1 (1.59)	6.55 (2.11)	8.1 (1.25)
Motivation	6.9 (2.25)	6 (2.45)	6.9 (2.2)	7 (2.2)
Interestingness	7.1 (1.62)	7.2 (2.31)	6.6 (2.23)	6.95 (2.14)
Self-efficacy	6.85 (1.57)	5.6 (1.54)	6.5 (2.01)	5.75 (2.02)

## Data Availability

The data are available upon request to the corresponding author’s email.
